# Bird Migration Seen South of Gaza

**Published:** 1918-05-18

**Authors:** 


### NATURE NOTES FROM PALESTINE.

### II. Bird Migration Seen South of Gaza.

I am in the desert in sight of the coast on to which the great fish spewed Jonah. Near by is where fascinating, importunate Delilah loved and sacrificed her lover at her country's call. Not far is the city whose gates strong-jointed Samson heaved on his massive shoulders. It is a desert now, though they that came earlier tell that then this dusty waste, across which sand drifts like a mist and is grit in the mouth, was patched with spreads of crops such as Samson fired with the foxes.

My camp is on an undulation, one of many such as it, but distinguished by the presence of a tree, the only tree near, not large, many an orchard apple tree would top it, but a centre of interest.

Perhaps because it is solitary and conspicuous this tree is a caravanserai of birds passing through the desert. It is beyond my knowledge to name the sorts I have seen in it, but not less than twenty-five species have passed a night in its shelter during the first three weeks of October. Four times I have found a corncrake beneath it, and once, at sunset, I almost saw the bird fly in. I was beneath the tree watching a flycatcher on a strand of barbed wire, when suddenly I was aware out of the corner of my eye that something had moved. Turning my head I saw a landrail on the bare sand six or seven yards to my right. The sand is absolutely bare, not a blade on it, so I do not think the bird had been lying there unnoticed by me; the plumage is not markedly protective in pale sand. All the landrails that have come in have been tame, though a little shy. Probably none of them know man to fear him. They walk about round and about the tree and two motor-cars kept in its shade, watching the watcher, and rather like to keep something between them and him. The surroundings must be very strange to them. In place of a great treeless plain, bare and desolate of man, there is a vast space spread with tents, huts, dumps of stores, and bivouacs sheltering thousands. A railway comes from Egypt and branches. Roads made sound for motor-cars by a pavement of wire netting cut across the desert. Great guns thunder, calibrating in preparation for great events. Air-planes wheel and hoot overheard practising communication work. Nothing is as it was but the eternal sky, the changeless changing sea, and the glory of dawn and sunset; yet the birds come and go undisturbedly upon their ancient way.

The most of the birds are wagtails, warblers, and willow wrens. The last shall be first. I certainly see two species, perhaps four. Of them one is the smallest bird I have ever seen alive, a tiny creature seemingly smaller than the "goldy-crest." I think I can differentiate at least twelve warblers. The naming is beyond me and I am not clear of the difference between willow wren and warbler, so I will lump the lot and say that I have seen some fifteen or sixteen kinds of small birds characterised by slender bills, very slender legs, small size, and great alertness in pursuit of insect-life amongst the bushes. The sedge-warbler, the blackcap, a white-throat, not the common nettle-creeper, a willow wren I know in England, and the tiny little fellow are some of them.

There are always flycatchers by the tree of a kind very like, if not identical with, the spotted flycatcher of English gardens. The difference in the ways of the warbler group and the flycatcher group is striking. The flycatchers sit on a wire fence, a derelict tent-peg, or a piece of dead branch fallen from the tree, and sally to take insects in the air. The warbler group seldom leaves the tree, but works ceaselessly and industriously along the twigs and through the leaves searching for and catching insects. What it is they find to live on beats me. Actually in the tree I can find nothing but house flies, and of them not many, and occasional other insects chiefly of the wasp and bee sorts. Humming-bird hawkmoths frequent the tree and hover before the small blossoms sticky with some sweetness secreted I think from the flowers; but it may be a honey-dew, not a honey. It is equally hard to conceive how the flycatchers make meals. There is a small grasshopper, quite minute and so extraordinarily like in shape and colour to a drifting grass-seed that I seldom find one, and never except from the accident of moving it as I walk, a few beetles, scarabs too large for the small birds to tackle even if they fancied them for food, a black and white hunting beetle, and a small variety of sad-coloured moths that fly by night to the light in my tent. This list comprises nearly all the insects I know to exist here. None the less I watch the flycatchers fly out time and again and seize something, and the wagtails actively chasing across the sand, springing up at things invisible to me or picking off equally invisible things from drifts of dry chaff and the scarce sprouts of wiry grasses.

The wagtails I suppose to winter here. They are in such numbers that literally hundreds now roost of nights in the tree, and their number has increased since I arrived. Their ways are different from the ways of the warblers, and compared to them they are giants. As the sun sets they begin to gather at the tree and perch close about it on the sand and pass a little interval before bed-time snatching any morsel that may serve to fill a hungry corner. Such corners should be many, a man might think, but that the birds look well-fed and hearty. As the sun sinks more and more come in; the party gets into the tree about the time the last of the sun's rim is hidden by the rim of the desert. Getting in is a great business, for the lodgers are many, the places not unlimited, and already the travellers have taken some. So there is much fluttering about and flying out and flying in again. Sometimes there is a sudden slight alarm and all whirr out explosively. It is as when a gust blows autumn leaves off a beech tree and sends them whirling above it to see the hundreds of birds fluttering round the roost. With morning the wagtails leave and spread themselves broadcast over the waste. In all directions, in ones and twos, and threes and fours, little parties, seldom over six or seven together, are encountered hunting over the sand for food. Again what is got is a mystery—flies; creatures from dung; scraps off bones, but this is a scarce supply because of the care taken to burn all organic refuse; grasshoppers. Though there seems almost no food at all, yet hundreds of wagtails are collecting a living. This sort is a pied wagtail, but often with them up to the third week of the month were two sorts of yellow wagtails. I do not see them now. I suppose they have passed on.

The warblers arrive at the tree in such way that it always appears to me that the number steadily grows greater all day and is at greatest an hour or so

☛ The previous article appeared on April 20, page 53.

### NATURE NOTES FROM PALESTINE—(*continued*).

before sunset. The number varies from day to day. I think that the birds as they come to the tree drop into it and settle down for the remainder of the day and the night. With them and the wagtails often three or four hundred birds must sleep in the shelter of its leaves. As twilight fades the tree becomes full of thousands of little ticking, clicking noises curious to hear, almost musical and suggestive of fairies. It is the birds making toilet, preening and arranging feathers before they tuck themselves up for the night.

Many redstarts come in, and a bird like a robin with a blue throat. Twice I have seen a solitary starling; twice a bird the size and shape of a blackbird, of dull plumage, nearly black, shy, and careful to keep a branch between it and the curious. I think it may be the thrush-like bird that whistles sweetly in the broken cliffs at the north end of Malta. Two or three red-backed shrikes of this year's hatch have spent nights in the tree, and once I saw a grey shrike larger than they, a sort often seen in Macedonia. One day I saw another of these last as I rode in the desert here, and one day a fine specimen of an adult cock red-backed shrike. Wheatears are common, scattered about the desert and often near the tree, but it is not their way to perch on it. Swallows often visit it, not to roost, but to hawk about it for a little space. I see many swallows, solitary or in small parties. When I am riding, often some of these birds join me and keep with me for miles to take the insects my horse's feet disturb. And so, though we start solitary, after a little while my horse and I are two of a little company that grows as we go till we may be a dozen. The birds are so bold and eager in pursuit that sometimes one will brush the horse's head with a wing, and often one will pass beneath its neck. Though they hunt thus near to me, their prey is so small that I have never yet detected it.

It is interesting that none of the birds that come to the tree show signs of tiredness, nor of distress from exertion. All are dapper and alert as little birds should be, and never rest, except by night, from search for food. Either their journey has not been long, or a long journey does not weary them. Their habitual restlessness may explain absence of fatigue. From dawn to sunset none is ever still. They creep and hop and fly and flutter about the branches ceaselessly. They remind me of children playing in a garden hour after hour, running up and down the paths, in and out the bushes. So much motion, if continuous in one direction, would take both sorts of little things far.

It is strange I never see the birds arriving. Perhaps it is because they are so small that at a very few hundred yards they would be out of sight, and so swift that the time any would be in view from point of coming into sight to time of making the tree must be very short. Moreover, it is only at intervals and for short times that I can stay to watch them. All day the number seems to increase, so I imagine them coming in by ones and twos and little parties. There is a steady flow through the tree. I think some go on the next morning, others rest a day or two, perhaps longer, but the stream never ceases. Sometimes it floods the tree, sometimes it slacks to a dozen or so of birds. Departure is early in the morning, but I sleep so soundly that I am never awake to see it. The most are gone before I open my eyes, and then there remain only the few that mean to pass the day in the tree, and odd stragglers visiting and even venturing into the tents in search of a light breakfast of sleepy flies to support them on their way.—October 1917.

*By a Staff Officer, Army Medical Service, and the Author of "Nature Notes."*

